# External validation of a prognostic model predicting renal graft function one year after brain-dead donor kidney transplantation

**DOI:** 10.1007/s00423-025-03962-8

**Published:** 2026-02-05

**Authors:** Philipp Tessmer, Clara A. Weigle, Franziska A. Meister, Bengt A. Wiemann, Wilfried Gwinner, Anja Mühlfeld, Rafael Kramann, Dennis Kleine-Döpke, Nicolas Richter, Felix Oldhafer, Florian W. R. Vondran, Harald Schrem, Oliver Beetz, Ulrich Zwirner

**Affiliations:** 1 Department of General, Visceral, Pediatric and Transplant Surgery, University Hospital RWTH Aachen, Aachen, Germany; 2 Department of Nephrology and Hypertension, Hannover Medical School, Hannover, Germany; 3 Division of Nephrology and Immunology, University Hospital RWTH Aachen, Aachen, Germany; 4 Department of General, Visceral and Transplant Surgery, Hannover Medical School, Hannover, Germany; 5 Department of General and Visceral Surgery, Klinikum Chemnitz, Chemnitz, Germany

**Keywords:** Deceased donor kidney transplantation, External validation, Renal graft function, Kidney transplantation, Prognostic model

## Abstract

**Purpose:**

A German transplant center recently published a prognostic model predicting graft function one year after deceased donor kidney transplantation (KTx) relying on pre-transplant variables. The aim of this study is to externally validate this model.

**Methods:**

We retrospectively analyzed clinical data from deceased donor KTx recipients undergoing transplantation between January 2007 and December 2023 at University Hospital Rheinisch-Westfälische Technische Hochschule (RWTH) Aachen. Receiver operating characteristics (ROC) curves were analyzed to validate the prognostic model based on donor age, donor serum creatinine, recipient body mass index, re-transplantation > 2nd KTx, and cold ischemia time. Glomerular filtration rates were categorized using the Kidney Disease: Improving Global Outcomes (KDIGO) categories G1 – G5.

**Results:**

A total of 494 kidney transplantations were performed at our institution, 350 (70.9%) thereof from donation after brain death. The median one-year estimated glomerular filtration rate (eGFR) was 42 [12–94] mL/min/1.73 m^2^. A validation for all eGFR categories was only possible with recalibration of the constant and coefficients of the original model, whereas without recalibration it could only be validated for KDIGO G2 and G4. Unfavourable recipient/donor-pairings with eGFR categories G4 and G5 one year after KTx could be predicted with an area under the ROC curve (AUC) > 0.700 in the validation and the original study cohort.

**Conclusion:**

We successfully validated the prognostic model for prediction of eGFR categories G4 and G5, which is of high clinical importance to identify outcomes with marginal graft function one year after KTx, thereby facilitating the avoidance of futile recipient/donor-pairings during allocation.

**Supplementary Information:**

The online version contains supplementary material available at 10.1007/s00423-025-03962-8.

## Introduction

Kidney transplantation (KTx) is the treatment of choice for end-stage renal disease, as transplant recipients benefit from superior patient survival [1–3] and improved quality of life [4] compared with dialysis patients. In Germany, there is a large disparity between organ demand and supply resulting in median waiting times of approximately nine years within the Eurotransplant kidney allocation system (ETKAS) [5]. Medical factors are of paramount importance when selecting an optimal kidney transplant recipient for a specific donor organ to ensure adequate early organ function [6]. One-year creatinine levels [7] and one-year estimated glomerular filtration rate (eGFR) [8] are suitable indicators for long-term graft survival. Zwirner et al. recently published a prognostic model predicting eGFR categories according to the Kidney Disease: Improving Global Outcomes (KDIGO) guidelines one year after deceased donor kidney transplantation, relying solely on variables available before transplantation [9]. The training data set consisted of 1.172 kidney transplants from donation after brain death (DBD) donors between January 2000 and December 2012 in Hannover, Germany [9]. If this model is valid to external cohorts, it could be suitable to predict KDIGO-based eGFR categories one year after KTx, thereby serving as an easy-to-apply prognostic tool for the prediction of long-term graft survival, without the need for histological analyses via pre-transplant biopsies. The model could be used to optimize kidney allocation, improve recipient/donor-pairing and thus to decrease the need for re-transplantation after failure of the first graft. The aim of this study was to externally validate the proposed prognostic model in our validation cohort of DBD kidney transplantations from Aachen, Germany.

## Materials and methods

### Study design

**Fig. 1:**
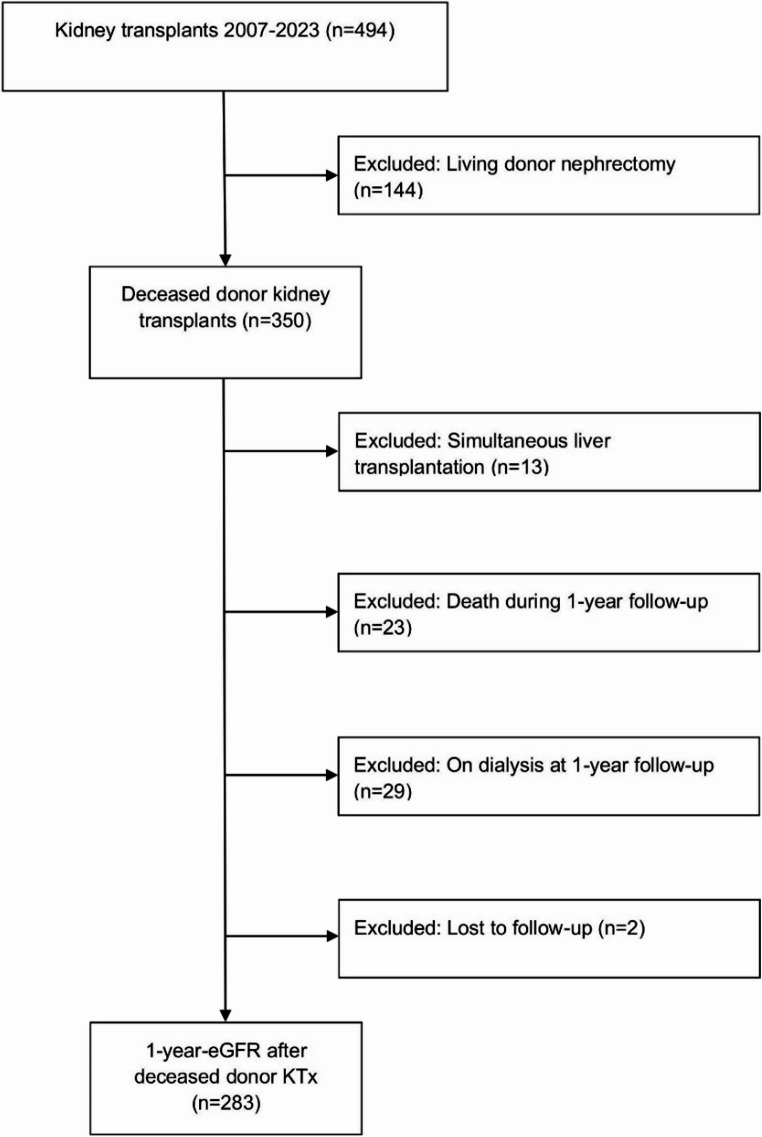
Study flow chart. Overview of excluded and included patients for later analysis. The figure was created with adobe photoshop CS4 (Adobe Systems Incorp., San Jose, CA, USA)

This external validation study was conducted according to the Transparent Reporting of a multivariable prediction model for Individual Prognosis or Diagnostics (TRIPOD) guidelines [10]. We retrospectively analyzed clinical data from all patients undergoing DBD kidney transplantation at the University Hospital Rheinisch-Westfälische Technische Hochschule (UH-RWTH) Aachen between January 2007 and December 2023. Clinical data included donors’ and recipients’ baseline characteristics, laboratory parameters and transplant data. The parameters that were identified as significant in the multivariate logistic regression model of the aforementioned publication [9] were used as test variables in Receiver Operating Characteristic (ROC) analyses. The included parameters were donor age, last donor serum creatinine, recipient body mass index, re-transplantation > 2nd kidney transplantation, and cold ischemia time (CIT). Follow-up was one year after kidney transplantation (± 3 months). Patients who returned to dialysis at the time of follow-up were excluded from the analysis to ensure compatibility with the original study cohort. Patients with living kidney donation or simultaneous liver or pancreas transplantation were also excluded. Fig. 1 depicts the flow of patients through the study. In the first year after kidney transplantation, all patients received a standard triple immunosuppressive regimen with tacrolimus, mycophenolate-mofetil and methylprednisolone after induction therapy with basiliximab. Patients with > 5% missing values (*n* = 2) were excluded from the study.

### Study endpoints

The study endpoint used for the validation of the prognostic model published by Zwirner et al. [9] was the recipient’s graft function according to KDIGO-based eGFR categories one year after kidney transplantation (± 3 months).

### Definition of variables

As in the original publication [9], the glomerular filtration rate of the kidney transplant was estimated using the 4-variable Modification of Diet in Renal Disease (MDRD) formula as published by Levey et al. [11]. eGFR values were categorized using the KDIGO guidelines [12]. The degree of albuminuria was not taken into account as a factor for categorization of renal function.

### Surgical procedure

In the majority of cases, heterotopic graft implementation to the right or left iliac fossa was performed. Arterial and venous anastomoses were preferably applied to the common and external iliac artery and vein, respectively. In most cases, modified Lich-Gregoire [13, 14] ureteroneocystostomy was performed, with temporal stenting of the graft ureter.

### Statistical analysis

Statistical significance was set at a two-sided *p*-value < 0.050. Descriptive statistics were conducted using SPSS statistical software. The original cohort from Hannover and the external validation cohort from Aachen were compared in regard to the distribution of model variables using the two-sided Mann-Whitney-U-test after confirmation of nonparametric data distribution with the Shapiro-Wilk-test (SPSS Statistics 29.0.1.0, IBM Corporation, Armonk, NY, USA). Nominal variables were tested with the two-sided Chi-square test.

Initial ROC analyses were conducted with the intention to externally validate the previously published model from Hannover [9] using JMP Pro 13 software (SAS Institute, Cary, NC, USA). These initial analyses were performed with recalibration and thus reweighing of the coefficients and the constant of the original multivariable ordinal regression model. Areas under the receiver operating curve characteristic (AUC) analyses were used to test the performance and external validity of the prognostic model to predict each eGFR category one year after KTx in the validation cohort from Aachen. An eGFR category of G1 was set as reference category, indicating unimpaired renal graft function.

Further AUC analyses were performed to test validation without recalibration of the original model using MedCalc Version 23 (MedCalc Software Ltd, Ostend, Belgium). For these analyses we also included 95% confidence intervals (95%-CIs), best Youden indices and calibration plots (see supplementary figures).

Additionally, the prediction of the combined eGFR categories G4 and G5 in both, the original cohort from Hannover and the validation cohort from Aachen, was assessed in order to evaluate potential clinical usefulness of the original Hannover model without recalibration in both cohorts for the prediction of unfavourable recipient/donor-pairings.

### Ethical approval

 The Institutional Review Board of UH-RWTH waived informed consent due to the collection of routine clinical data and the retrospective study design. Prior to analysis, patient data and records were anonymized and de-identified.

## Results

### Descriptive statistics

In the observation period, 494 kidney transplants were performed at UH-RWTH. 144 (29.1%) grafts were from living and 350 (70.9%) from deceased donors. Of the 350 deceased donor kidney transplants, 67 were excluded for various reasons: returning to dialysis before reaching the study endpoint (*n* = 29; 43.3%), patient death (*n* = 23; 34.3%), simultaneous liver transplantation (*n* = 13; 19.4%) and loss to follow-up (*n* = 2; 3.0%). Thus, the eGFR of 283 deceased donor kidney transplant recipients at one-year follow up was finally analyzed.

In the study group, most recipients were male (*n* = 190; 67.1%) with a median age of 57 (22–80) years and a median body mass index (BMI) of 25 (15–52) kg/m^2^, respectively. The median donor age and the donor’s last serum creatinine was 57 (8–88) years and 88 (25–650) µmol/L, respectively. The median eGFR one year after transplantation was 42 (12–94) mL/min/1.73 m^2^. One-year eGFR was categorized as KDIGO G1, G2, G3a, G3b, G4, and G5 in 2 (0.7%), 39 (13.8%), 70 (24.7%), 107 (37.8%), 62 (21.9%), and 3 (1.1%) cases, respectively (see below). Most patients received their first or second graft (*n* = 279; 98.6%). Only four (1.4%) patients were third graft recipients. In our cohort, no fourth or fifth kidney transplants were performed. The median cold ischemia time was 743 (54-1760) minutes. Table 1 shows the patients’ descriptive statistics.

**Table 1:** Descriptive statistics of the validation cohort of n=283 DBD kidney transplant recipients from Aachen. Parameters that were identified as statistically significant in the multivariate analysis by Zwirner et al. are marked in italic. These parameters were then used as test variables in subsequent ROC analyses

Parameters	
Kidney transplants, n [%]	494 [100.0]
- Deceased donor, n [%]	350 [70.9]
- Living donor, n [%]	144 [29.1]
- Study group, n [%]	283 [57.3]
Recipient age, median [range]	57 [22–80]
Recipient sex	
- Male, n [%]	190 [67.1]
- Female, n [%]	93 [32.9]
Recipient 1-year-eGFR (ml/min/1,73 m²), median [range]	42 [12–94]
*Recipient BMI (kg/m*^*2*^*)*,* median [range]*	25 [12–52]
*Donor age (years)*,* median [range]*	57 [8–88]
*Donor last serum creatinine (µmol/L)*,* median [range]*	88 [25–650]
*Cold ischemia time (min)*,* median [range]*	743 [54-1760]
*Transplantation > 2nd KTx*,* n [%]*	4 [1.4]

*BMI* Body Mass Index, *eGFR* estimated glomerular filtration rate, *KTx* Kidney transplantation.

### Comparison of the study cohorts from Aachen and Hannover

The comparison of both donor and recipient characteristics revealed several statistically significant differences between the validation cohort from Aachen and the original study cohort from Hannover. The donors’ age and serum creatinine were each significantly higher in Aachen. Recipient BMI was also significantly higher in the validation cohort from Aachen, while the cold ischemia time was shorter in Aachen compared to Hannover. The only variable that did not differ significantly between the two centers was the proportion of patients undergoing kidney re-transplantation beyond the 2nd KTx. All corresponding statistical parameters and *p*-values are shown in Table 2.

**Table 2:** Comparison of all variables included in the predictive model between the validation cohort (Aachen) and the original study cohort (Hannover). Statistically significant differences were identified using the Mann-Whitney-U-test for continuous variables and the Chi-square test for binary variables

	Aachen					Hannover					
Continuous variables	**Mean**	**Median**	**Min**	**Max**	**SD**	**Mean**	**Median**	**Min**	**Max**	**SD**	***p***
Recipient BMI (kg/m²)	25,7	25,0	15,0	52,0	4,6	24,7	24,5	15,0	38,0	3,9	**0.004**
Donor age (years)	56	57	8	88	15	50	52	5	88	16	**< 0.001**
Donor last serum creatinine (µmol/L)	119	88	26	650	83	103	80	18	725	87	**< 0.001**
Cold ischemia time (min)	753	743	54	1760	246	930	877	127	2430	364	**< 0.001**
Binary variables											
Transplantation > 2nd KTx	yes = 4 no = 279					yes = 32 no = 1140					**0.201**

*BMI* Body mass index, *KTx* Kidney transplantation, *Min* Minimum, *Max* Maximum, *SD* Standard Deviation.

### Results of validation with recalibration

**Fig. 2:**
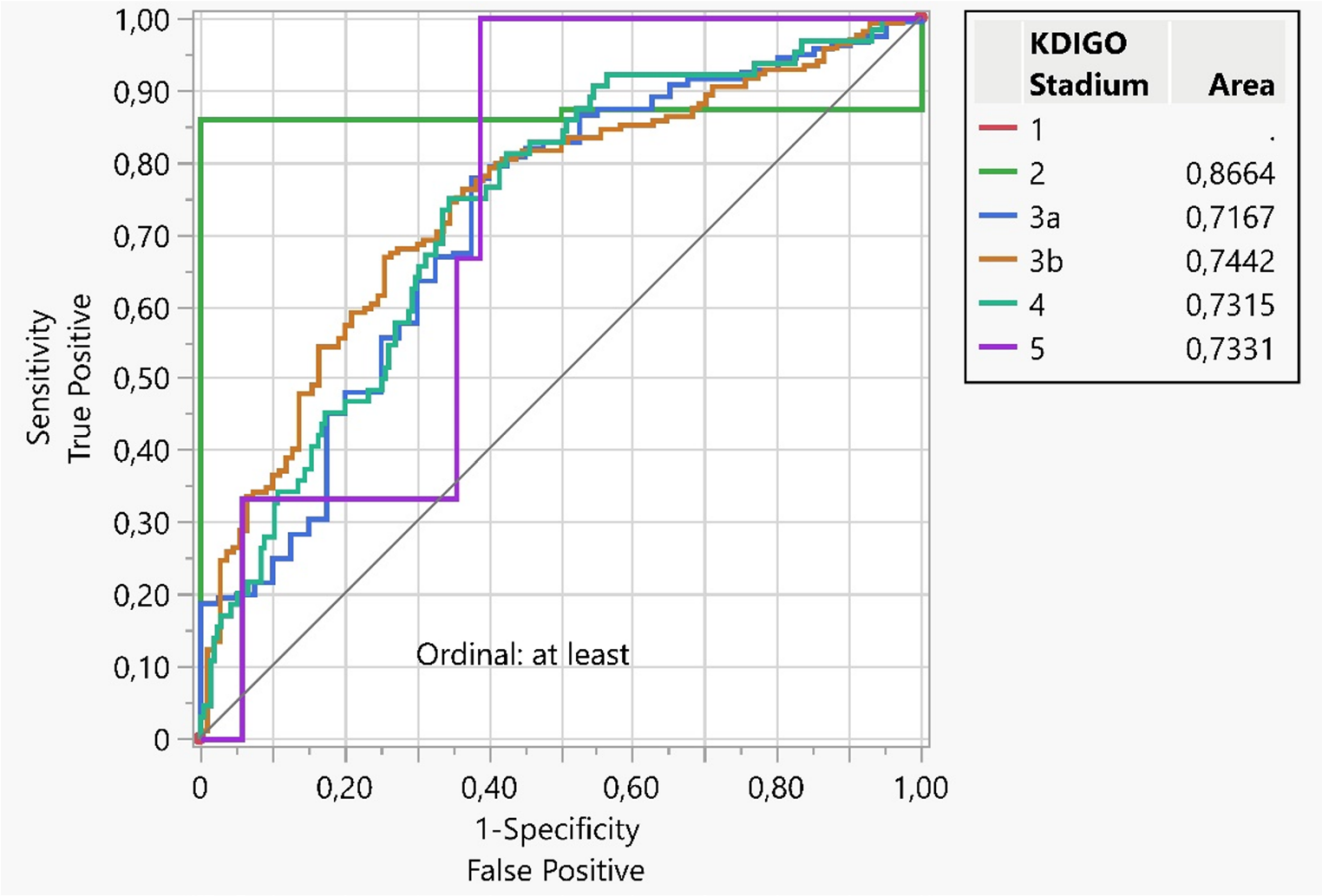
External performance of the proposed multivariable regression model to predict estimated glomerular filtration categories according to the KDIGO classification one year after DBD kidney transplantation. For each eGFR category, the corresponding area under the receiver operating curve is shown. KDIGO G1 was set as reference category. AUC>0.700 indicates a sufficiently high sensitivity and specificity of prediction. The figure was created with Jump Pro 13 software (SAS Institute, Cary, NC, USA) and optimized for publication using Adobe Photoshop CS4 (Adobe Systems Incorp., San Jose, CA, USA)

Using the proposed prognostic model by Zwirner et al., the AUC values for KDIGO eGFR categories G2, G3a, G3b, G4, and G5 were 0.866, 0.717, 0.744, 0.732, and 0.733, respectively, indicating a sufficiently high sensitivity and specificity of prediction for potential clinical application for each eGFR category with recalibration of the original model. Table 3 classifies the patients’ one-year eGFR categories according to the KDIGO classification. Fig. 2 shows the corresponding ROC curves for each eGFR category.

**Table 3:** One-year eGFR after deceased donor KTx was categorized according to the kidney disease: improving global outcomes guidelines (KDIGO) categories G1 to G5, *n* = 283

eGFR category	Number of patients [%]
G1	2 [0.7]
G2	39 [13.8]
G3a	70 [24.7]
G3b	107 [37.8]
G4	62 [21.9]
G5	3 [1.1]

### Results of validation without recalibration

**Fig. 3:**
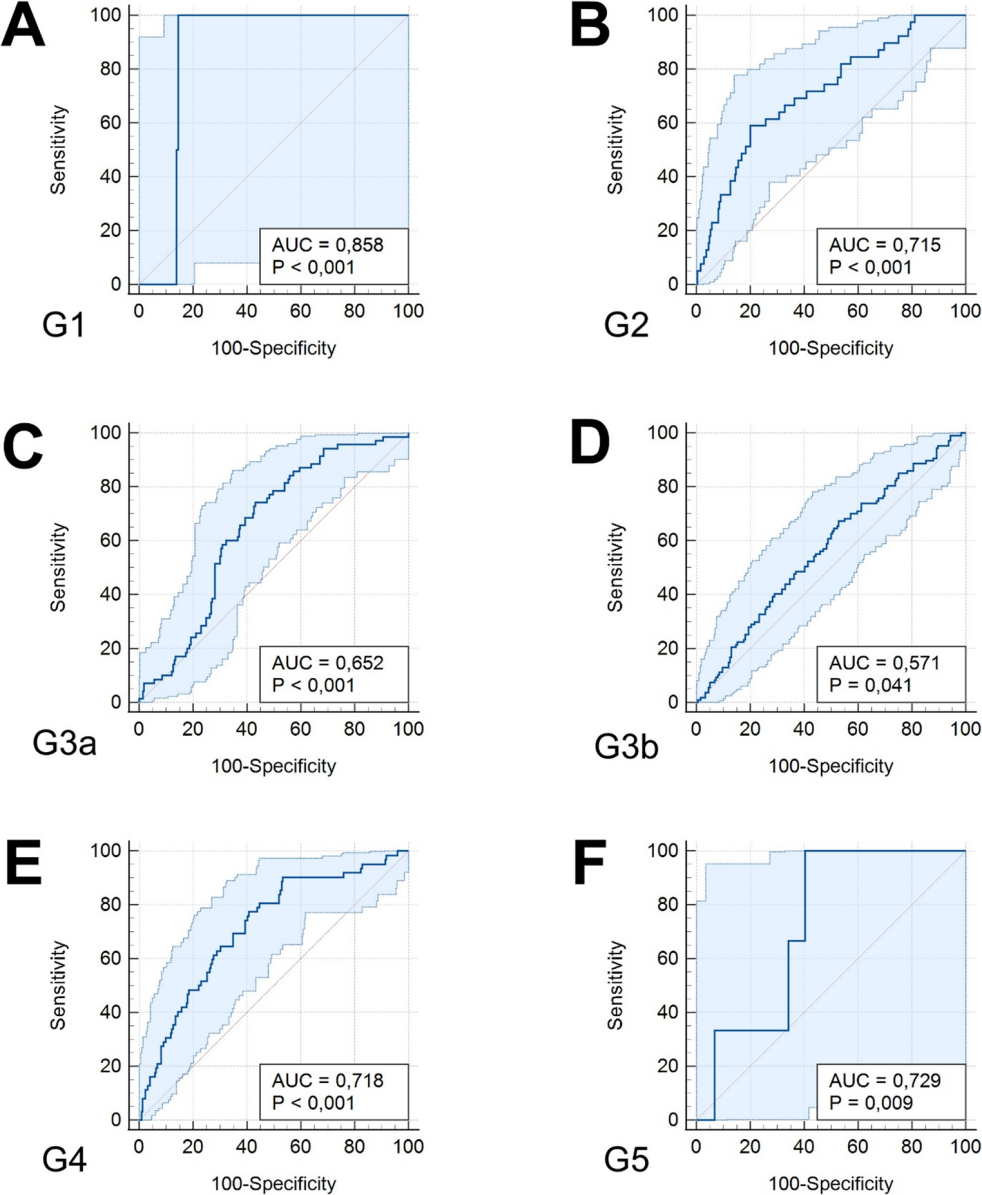
Results of the AUC analyses without recalibration including 95%-CIs for each estimated glomerular filtration category according to KDIGO from G1 (**A**) to G5 (**F**) in the validation cohort from Aachen one year after DBD kidney transplantation. The figure was created with MedCalc Version 23 (MedCalc software Ltd, Ostend, Belgium) and optimized for publication using Adobe Photoshop CS4 (Adobe Systems Incorp., San Jose, CA, USA). AUC area under the receiver operating curve

In order to prevent recalibration and to properly validate the prognostic model proposed by Zwirner et al., we performed a second AUC analysis for each eGFR category, applying the original formula for calculation of the linear logits as presented in [9]. AUC for KDIGO G1 was 0.858 (95%-CI: 0.811–0.896; best Youden index > − 3.057). AUC for KDIGO G2 was 0.715 (95%-CI: 0.658–0.767; best Youden index > − 3.396). AUC for KDIGO G3a was 0.652 (95%-CI: 0.594–0.708; best Youden index > − 3.904). AUC for KDIGO G3b was 0.571 (95%-CI: 0.511–0.630; best Youden index ≤ − 3.717). AUC for KDIGO G4 was 0.718 (95%-CI: 0.661–0.769; best Youden index ≤ − 3.646). AUC for KDIGO G5 was 0.729 (95%-CI: 0.673–0.780; best Youden index ≤ − 4.117). Fig. 3 shows ROC curves for each eGFR category with 95%-CIs. In addition, calibration plots indicating sensitivity and specificity for each eGFR category with corresponding 95%-CIs are shown in Supplementary Fig. 1.

### Prediction of a combined eGFR category G4 + G5 group in both cohorts without recalibration

**Fig. 4:**
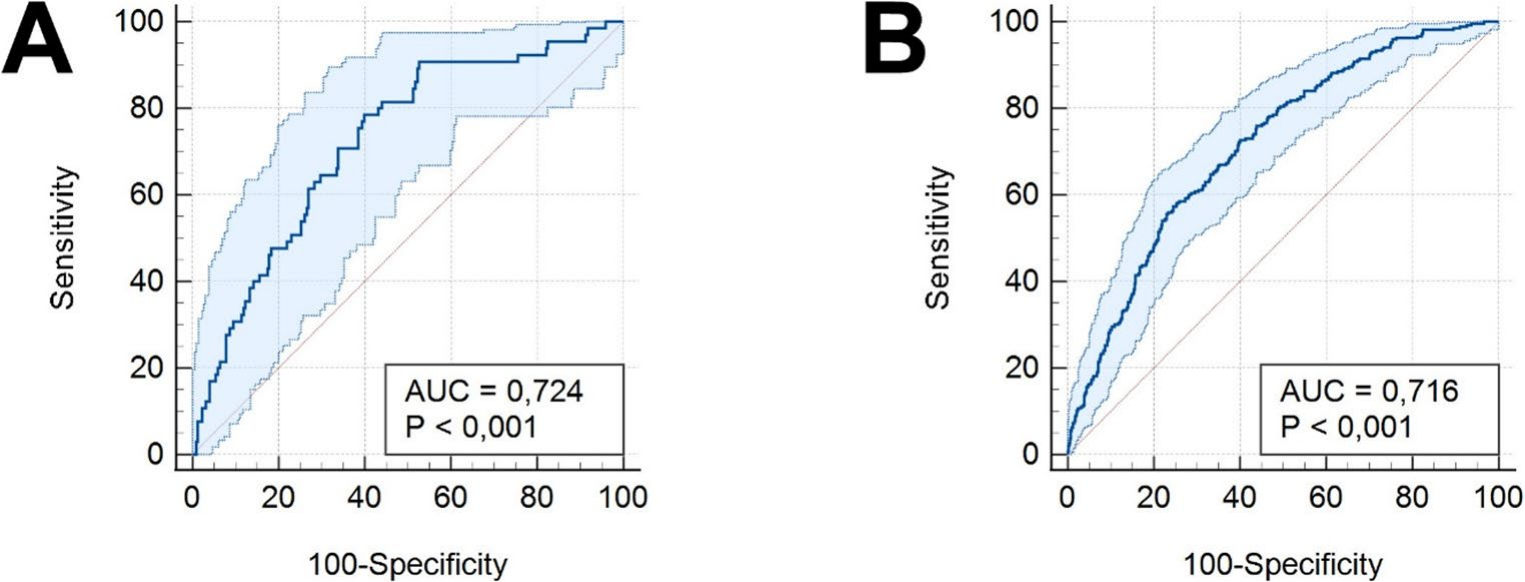
Results of the AUC analyses without recalibration including 95%-CIs for a combined group of eGFR categories G4 and G5 one year after DBD kidney transplantation in the validation cohort from Aachen (**A**) and in the original study cohort from Hannover (**B**). The figure was created with MedCalc Version 23 (MedCalc software Ltd, Ostend, Belgium) and optimized for publication using Adobe Photoshop CS4 (Adobe Systems Incorp., San Jose, CA, USA). *AUC* area under the receiver operating curve

For a prognostic assessment of unfavourable KTx recipient/donor-pairings, i.e., eGFR categories G4 and G5 one year after KTx, we assessed the performance of the prognostic model without recalibration for all patients in our study cohort and compared it to the original cohort from Hannover University Hospital. AUC for KDIGO G4 + G5 was 0.724 (95%-CI: 0.668–0.775; best Youden index ≤ − 3.909) in Aachen (Fig. 4 A). In contrast, AUC for KDIGO G4 + G5 was 0.716 (95%-CI: 0.689–0.742; best Youden index ≤ − 4.053) in Hannover (Fig. 4B). Additionally, calibration plots indicating sensitivity and specificity with 95%-CIs are shown in Supplementary Fig. 2.

## Discussion

This study aimed to evaluate the external validity and performance of a recently published prognostic model predicting KDIGO-graded renal graft function one year after DBD kidney transplantation. For reliable prognostic models, areas under the ROC curves typically range from 0.600 to 0.850 [15], with values above 0.700 generally considered to reflect sufficiently high predictive sensitivity and specificity in a clinical context [16]. With the initial findings of our study, we were able to demonstrate AUC values above 0.700 for each KDIGO-based eGFR category in our cohort of DBD kidney transplant recipients. Nevertheless, this came with two obvious limitations: Firstly, the prediction of eGFR categories G2 and G5 one year after KTx could not be reliably validated due to a very small number of patients in both categories within the validation cohort. Furthermore, this validation attempt across all eGFR categories relied on recalibration of the original model. In order to exclude the recalibration effect in the validation process, we performed additional AUC analyses applying the original formula as described by Zwirner et al. [9]. Without recalibration, the prognostic model could be validated for KDIGO G2 and G4, nonwithstanding the statistically significant differences between the validation and the original study cohort (Table 2), whereas a validation for eGFR categories G3a and G3b was not possible. The validation of the prediction of KDIGO G1 and G5 one year after KTx without recalibration was not convincing, despite showing AUC values > 0.700, due to very small numbers of patients in these subgroups.

Interestingly, the originally published model from Hannover was able to predict only residual graft function (eGFR categories G4 and G5) one year after KTx without recalibration (AUCs > 0.700) in both cohorts from Aachen and Hannover. This indicates potential clinical usefulness of this model to avoid unfavourable recipient/donor-pairings by the prediction of poor outcomes. Reallocation of such donor organs to recipients with low BMI and no need for re-transplantation beyond the 2nd KTx would likely avoid outcomes with only residual graft function one year after KTx. The robustness of this finding is fostered by the fact that the distribution of all but one variable of the original model was significantly different between the cohorts from Aachen and Hannover. However, KTx followed by either death or return to dialysis within one year as the most unfavourable outcomes could not be investigated in this validation study because these cases were excluded in the original study [9].

In total, 1172 kidney transplant recipients from the original study cohort and 283 patients from our cohort were included, resulting in an overall sample size of 1455 patients for the development and validation of the prognostic model. In addition to this comparatively large sample size, the inclusion periods of the original cohort (01/00–12/12) and our cohort (01/07–12/23) span a long-term period of 24 years in total. Despite these different inclusion periods and thus, independent of the time of kidney transplantation, the model was successfully validated for prediction of recipient/donor-pairings with marginal graft function one year after KTx. Since the model was developed using a northern and validated in a western German cohort, it is likely to be robust against center-specific biases. Therefore, it can be assumed that the model likely has a more general applicability across transplant centers. In addition, the prognostic model has the obvious advantage that all required parameters are easily available before the actual KTx surgery, with exception of the CIT, which can be estimated with sufficient accuracy during the allocation process. In conclusion, it should be feasible to easily implement the model into the clinical routine, although additional centers and KTx cases have to be included in subsequent validation studies before a change of the allocation procedure becomes reasonable. To date, a comparable model has only been published for the prediction of pancreas and kidney function following simultaneous organ transplantation [17].

Most limitations of this validation study are attributable to the comparatively small size of the validation cohort. This was especially apparent for eGFR categories G1 and G5. An inclusion of patients with the worst outcomes - death or return to dialysis during the first 12 months after KTx – into the analysis might not only increase the predictive value of the resulting prognostic model, but could also lead to the identification and thereby avoidance of futile recipient/donor-pairings during allocation. However, this question will be addressed in a subsequent study, as it was not within the scope of the original work by Zwirner et al. and therefore also not part of the current validation study.

Based on our study results, the prognostic model appears applicable within Germany; however, its validity in other countries participating in the ETKAS cannot be assumed without further international validation. Furthermore, we used eGFR categories – an ordinal scale – as state variables in the ROC analysis. The predictive accuracy is therefore inferior to continuous variables, such as specific eGFR values. Nevertheless, previous predictive models only rely on nominal study endpoints, for example one-year graft loss [18]. Using eGFR categories offers practical advantages for clinical application, as the KDIGO guidelines are the most common classification system for chronic kidney disease and are widely accepted by nephrologists, transplant surgeons and other healthcare organizations across the globe [19]. In the original study population by Zwirner et al., eGFR was calculated using the MDRD formula [9]. To ensure a maximum comparability, we also used the MDRD formula for eGFR calculation. The Chronic Kidney Disease Epidemiology Collaboration (CKD-EPI) formula was shown to be superior in accuracy compared to the MDRD formula [20]. However, the majority of kidney transplant recipients (242/283 = 85.5%) in our cohort had an eGFR below the reported threshold of 60 mL/min/1.73 m^2^, above which the MDRD equation lacks accuracy [20]. Therefore, the accuracy of the eGFR estimation in our study should be sufficiently precise.

In the past, several prognostic scoring systems were developed to predict graft outcomes after deceased donor KTx. These are summarized in Table 4. Firstly published in 2009, the kidney donor risk index includes donor and transplant variables to estimate the relative risk of graft failure compared to a 40-year-old healthy donor [21]. Its derivative, the kidney donor profile index, is based on the same variables and translates this relative risk into percentile ranks, aiming to simplify the score for clinicians [22]. However, experts have raised concerns that the predictive power of the kidney donor profile index is limited: In the original US-American publication, the average c-statistics were reported as 0.62 [21]; a similar value was found in an external validation study in a European cohort [30]. Thus, similar kidney donor profile indices cannot reliably predict outcome differences of specific kidney grafts. Additionally, since the kidney donor profile index only indicates the risk of graft failure relative to a healthy reference donor, it does not allow for predictions of graft survival at specific time points. This is an important limitation in comparison with the model by Zwirner et al., which does not predict nominal outcome parameters such as graft failure, but rather could predict KDIGO-based eGFR categories as an ordinal variable at a specific time point [9]. Since the one-year graft function was shown to predict long-term graft survival [7], the model by Zwirner et al. could serve as a surrogate parameter in this regard.

**Table 4:** Comparative overview of clinically established scoring systems for the prediction of different outcomes after KTx including functional graft performance, delayed graft function and graft loss

Score/System	Predicted Outcomes	Key predictors	Reported performance	Original reference
Hannover Model by Zwirner/Schrem et al.	KDIGO eGFR categories one year after DBD kidney transplantation	Donor age, donor creatinine, recipient BMI, cold ischemia time, ReTx > 2nd KTx	Without recalibration: KDIGO G2: AUC = 0.715 KDIGO G4: AUC = 0.718 KDIGO > 3b: AUC = 0.724	[9]
2-Step Score	DGF and 1-year death-censored transplant loss	DGF: cold ischemia time (CIT), donor & recipient body mass index, dialysis vintage, HLA-DR mismatches, recipient CMV IgG, donor age; Step 2: adds procurement biopsy/nephropathology 1-year-transplant loss: CIT, HLA-A, -B, -DR mismatches, donor age	DGF: c-statistics 0.672 (training), 0.704 (validation); 0.696 and 0.701 with nephropathology. 1-yr transplant loss: 0.700 (training), 0.769 (validation); 0.706 and 0.765 with nephropathology.	[18]
KDRI/KDPI	Relative risk of graft loss or death after deceased donor kidney transplantation compared to a healthy, 40-year-old, male reference donor (KDRI = 1.0)	Donor age, race, hypertension, diabetes, serum, creatinine, cerebrovascular cause of death, height, weight, DCD, HCV, HLA-B & DR mismatch, CIT, en-bloc/double transplant	5-year graft survival highest (> 1.45) vs. lowest (< 0.79) KDRI quintile: 63% vs. 82%	[21, 22]
ETKAS	Allocation system	Waiting time, HLA mismatches, pediatric priority, donor/recipient geographic/age criteria	No classic prognostic model, reported are improved waiting time and HLA matching	[23]
Irish DGF Nomogram	DGF after deceased donor kidney transplantation	Cold ischemia time, donor creatinine, body mass index, donation after cardiac death status and donor age.	AUC 0.704	[24]
Chapal DGFS	Delayed graft function	Cold ischemia time, donor and serum creatinine, recipient body mass index, induction therapy	AUC 0.73	[25]
Deceased Donor Score	1-year graft function and 6-year graft survival	Donor age, hypertension, creatinine clearance, cause of death, HLA-mismatches	Best performance grade C or above, not further specified	[26, 27]
iBox	Graft loss	eGFR, proteinuria, DSA, time post transplantation, four histological features	C index 0.81, 95% CI (0.79–0.83)	[28]
5-Year Risk Score (UK/France/Canada)	Predict 5-year graft failure	Recipient age, sex, race, acute rejection, transplant function at 12 months, albumin, proteinuria	Death-censored transplant failure: C statistics 0.78–0.90; overall: 0.75–0.81	[29]

*KDIGO* kidney disease: improving global Outcomes, *eGFR* estimated glomerular filtration Rate, *DBD* Donation after brain Death, *DCD* Donation after cardiac Death, *ReTx* re-transplantation, *KTx* kidney transplantation, *DGF* delayed graft function, *CIT* cold ischemia time, *HLA* human leukocyte antigen, *CMV* cytomegalovirus, *HCV* hepatitis C virus, *KDRI* kidney donor risk Index, *KDPI* kidney donor profile Index, *ETKAS* Eurotransplant kidney allocation System, *DGFS* Delayed graft function Score, *DSA* donor-specific antibody, *BMI* body mass index, *AUC* areas under the receiver operating curve characteristic

The deceased donor score is a donor-derived score that calculates one-year graft function and six-year graft survival after deceased donor kidney transplantation [26, 27]. It is more commonly used in Europe, particularly in Spain [27, 31] and the United Kingdom [32]. While the deceased donor score incorporates the number of human leukocyte antigen mismatches into the analysis, further recipient variables, e.g. the recipient’s age or the number of previous kidney transplants, are not considered [26] – a known limitation of the score. In contrast, the prognostic model by Zwirner et al. takes recipient variables such as the recipient’s body mass index and the re-transplantations beyond the 2nd KTx (thereby functioning as a surrogate parameter for various immunological risk factors) as well as transplant-specific variables like CIT into account [9].

 The standard Eurotransplant kidney allocation system (applying for recipients < 65 years) prioritizes the immunological matching of donor and recipient as well as ethical considerations such as medical urgency and waiting time over the use of donor-derived scores [23, 33]. Thus, a suboptimal donor-recipient matching with respect to the predicted recipient’s graft and patient survival is considered a limitation, thereby potentially decreasing overall organ utility [34].

Recently, Ernst et al. proposed a new scoring system to predict both delayed graft function (DGF) and one-year graft loss after brain-dead donor kidney transplantation [18]. Both scores by Zwirner and Ernst were developed and validated in German and European cohorts, respectively, using only brain-dead kidney donors as donation after cardiac death is forbidden in Germany according to the German Transplantation Law [35]. Nonetheless, there are some important differences when comparing the scores by Zwirner and Ernst. The number of patients included for the development of the prognostic model were *n* = 1172 by Zwirner et al. and *n* = 620 (for DGF) and *n* = 711 (for one-year graft loss) by Ernst et al., respectively. The number of patients in the validation sets, *n* = 158 (for DGF) and *n* = 162 (for one year-graft loss) by Ernst et al. and *n* = 283 used in this study, was different as well [18]. The Cologne score has the advantage of a comparatively high discriminative power, with c-statistics (each for training and validation set, respectively) of 0.67 and 0.70 for DGF and 0.70 and 0.76 for one-year graft loss. C-statistics slightly improved after taking the results of optional nephropathology (step two of the score) into account. This is superior to the kidney donor profile index [21] and will be interesting to be assessed in future (validation) studies. Furthermore, the 2-Step score is an easy-to-use tool with optional histopathology providing the opportunity to the allocating clinician in otherwise unclear cases [18]. The most important difference between the 2-Step-Score and the prognostic model by Zwirner et al. lies in the type of outcome variables they predict. The score by Ernst and colleagues estimates the risk of two nominal variables — specifically, delayed graft function and one-year graft loss — as major adverse events after kidney transplantation. In contrast, the prognostic model by Zwirner et al. predicts one-year eGFR categories, an ordinal variable, for a specific donor-recipient match [9]. Taken together, the 2-Step-Score offers a promising tool for kidney allocation within the ETKAS and may be superior to established scores. The now partially validated score by Zwirner et al. may serve as a valuable addition for the nephrologist or transplant surgeon responsible for organ allocation by improving individual decision-making, as it allows to predict one-year eGFR categories – especially G4 and G5 – in different recipients based on a specific donor graft.

We investigated and partially validated the prognostic model by Zwirner et al. that provides an accurate estimate of the eGFR category one year after DBD kidney transplantation using non-invasive and easy-to-obtain parameters all available before transplantation. This validation was only possible with recalibration for all eGFR categories, whereas it could only be validated for G2 and G4 without recalibration. Nevertheless, unfavourable KTx recipient/donor-pairings with an eGFR category of G4 or G5 one year after KTx could be predicted with sufficiently high sensitivity and specificity (AUC > 0.700) in the validation cohort as well as in the original study cohort. Thus, this prognostic model can improve recipient/donor-matching prior to KTx, thereby improving long-term graft survival, and may also be useful for the identification of recipients at risk for major adverse events. Such high-risk recipients could then be monitored more closely in follow-up care after KTx. Based on the results of our study, further validation studies in countries participating within the ETKAS are now warranted, before a broad application can become clinical reality.

## Conclusion

In this study, we partially validated the recently published prognostic model predicting KDIGO-graded eGFR categories one year after DBD kidney transplantation. Prediction of recipient/donor-pairings with marginal function (KDIGO G4 and G5) one year after KTx was possible with sufficiently high sensitivity and specificity. Before the model should be implemented in clinical practice to optimize kidney allocation, further and international studies with higher patient numbers and inclusion of the worst outcomes (death and early return to dialysis) are recommended.

## Supplementary Information

Below is the link to the electronic supplementary material.

Supplementary File (PDF 424 KB)

## Data Availability

The datasets used and/or analyzed during the current study are available from the corresponding author upon reasonable request.

## Abbreviations

95%-CI: 95% Confidence Interval
AUC: Areas under the receiver operating curve characteristic
BMI: Body Mass Index
CIT: Cold Ischemia Time
CKD-EPI: Chronic Kidney Disease – Epidemiology collaboration
CMV: Cytomegalovirus
DBD: Donation after Brain Death
DCD: Donation after Cardiac Death
DGF: Delayed Graft Function
DSA: Donor-Specific Antibody
eGFR: estimated Glomerular Filtration Rate
ETKAS: EuroTransplant Kidney Allocation System
HCV: Hepatitis C Virus
HLA: Human Leukocyte Antigen
KDIGO: Kidney Disease: Improving Global Outcomes
KDPI: Kidney Donor Profile Index
KDRI: Kidney Donor Risk Index
KTx: Kidney Transplantation
MDRD: Modification of Diet in Renal Disease
Re-Tx: Re-transplantation
ROC: Receiver Operating Characteristic
RWTH: Rheinisch-Westfälische Technische Hochschule
TRIPOD: Transparent Reporting of a multivariable prediction model for Individual Prognosis Or Diagnostics
UH-RWTH: University Hospital of Rheinisch-Westfälische Technische Hochschule Aachen
