# Supplementary material for: External validation of a prognostic model predicting renal graft function one year after brain-dead donor kidney transplantation
Source: Langenbecks Arch Surg. 2026 Feb 5;411(1):77. doi: 10.1007/s00423-025-03962-8 (PMC12891121; doi:10.1007/s00423-025-03962-8)
Supplement: Supplementary file 1 — Supplementary File (PDF 424 KB) [file 423_2025_3962_MOESM1_ESM.pdf]

Supplementary Figures

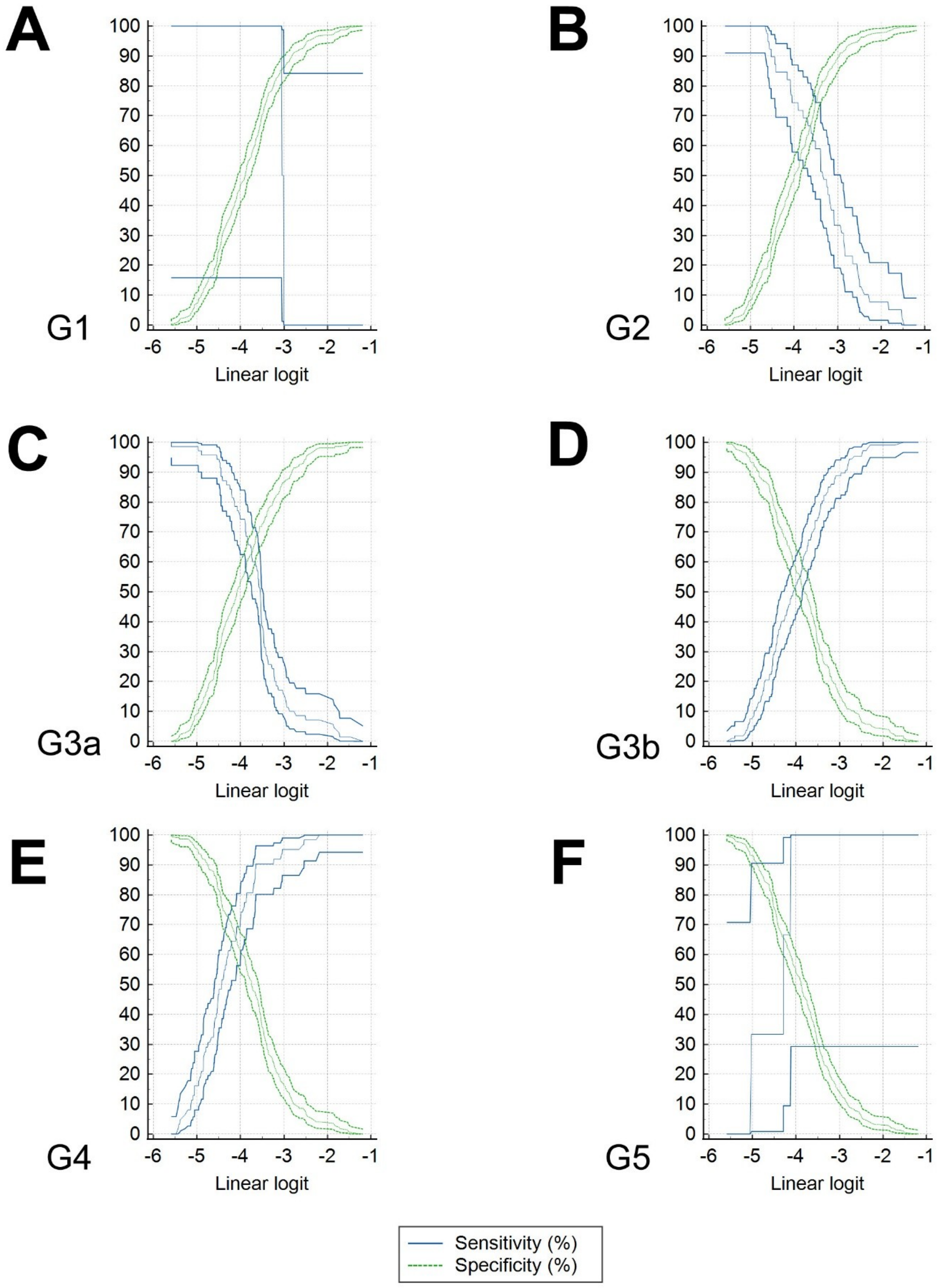

Supplementary Figure 1: Calibration plots including 95%-CIs for each AUC analysis performed without recalibration for each KDIGO eGFR category from G1 (**A**) to G5 (**F**) in the validation cohort from Aachen. The figure was created with

MedCalc Version 23 (MedCalc software Ltd, Ostend, Belgium) and optimized for publication using Adobe Photoshop CS4 (Adobe Systems Incorp., San Jose, CA, USA).

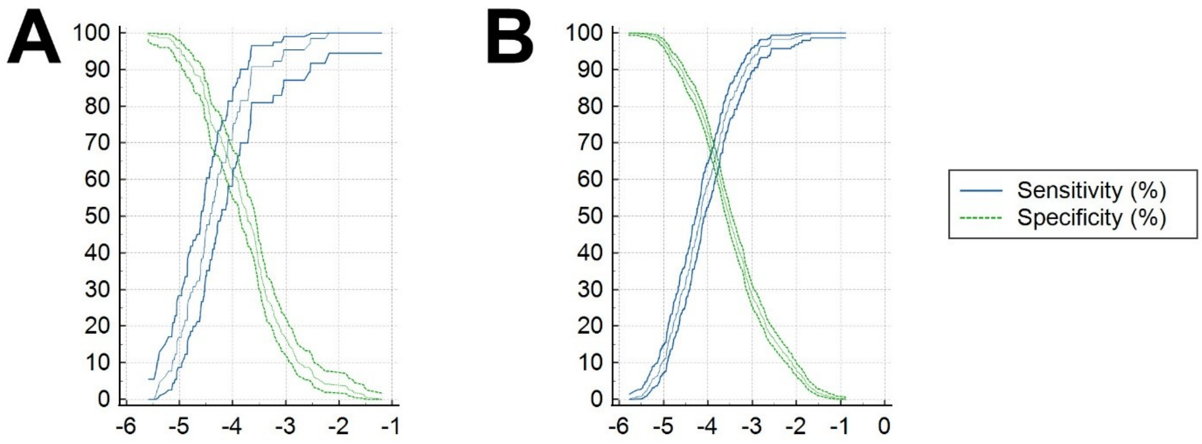

Supplementary Figure 2: Calibration plots including 95%-CIs for each AUC analysis performed without recalibration for the combined group of KDIGO G4 and G5 patients one year after DBD kidney transplantation in the validation cohort from Aachen (**A**) and in the original study cohort from Hannover (**B**). The figure was created with MedCalc Version 23 (MedCalc software Ltd, Ostend, Belgium) and optimized for publication using Adobe Photoshop CS4 (Adobe Systems Incorp., San Jose, CA, USA).
